# INVESTIGATING THE ROLE OF AHR IN MEDIATING SEX DIFFERENCES OF AGING MACROPHAGES

**DOI:** 10.1093/geroni/igz038.3080

**Published:** 2019-11-08

**Authors:** Evelyn Navar, Bérénice A Benayoun, Nirmal Sampathkumar, Jisoo Chae

**Affiliations:** 1 University of California at Davis, Davis, California, United States; 2 University of Southern California, Leonard Davis School of Gerontology, Los Angeles, California, United States

## Abstract

“Inflamm-aging” describes a state of chronic low-grade inflammation which occurs with age in the absence of infection. This process is related to many chronic age-related diseases. Aryl hydrocarbon receptor (Ahr), is a transcription factor that is thought to decrease inflammation, and decrease of Ahr with aging only in females was previously observed in a macrophage RNA-seq with aging. Based on this, I hypothesized that 1) Ahr expression will decrease with age in female cells; and 2) phagocytic activity and Ahr expression in macrophages will increase when exposed to estrogens (E2). To test these hypotheses, Ahr signaling was quantified by RT-qPCR in aging male and female mice BMDMs, and in macrophages that were treated with E2. I also performed a phagocytosis assay on macrophages treated with E2. I found a significant downregulation of Ahr in old female BMDMs. Ahrr (Ahr Repressor) was significantly downregulated in both old female and males with aging. Arnt (Ahr Nuclear Translocator) did not significantly change with aging. The qPCR performed on the E2 treated cells showed no significant trend for Ahr regulation. Finally, the phagocytosis assay revealed an overall increase in phagocytosis activity in cells treated with estrogen. Our hypotheses were supported by data showing a decrease in Ahr expression with age and increase in phagocytosis activity in estrogen treated cells. The RT-qPCR results for the E2 treated cells did not support our hypothesis, but could stem from a relatively short exposure time for estrogen.

